# Meridianin C inhibits the growth of YD‐10B human tongue cancer cells through macropinocytosis and the down‐regulation of Dickkopf‐related protein‐3

**DOI:** 10.1111/jcmm.13854

**Published:** 2018-09-24

**Authors:** Nam‐Sook Park, Yu‐Kyoung Park, Mahesh Ramalingam, Anil Kumar Yadav, Hyo‐Rim Cho, Victor Sukbong Hong, Kunal N. More, Jae‐Hoon Bae, David Bishop‐Bailey, Junko Kano, Masayuki Noguchi, Ik‐Soon Jang, Kyung‐Bok Lee, Jinho Lee, Jong‐Soon Choi, Byeong‐Churl Jang

**Affiliations:** ^1^ Department of Molecular Medicine College of Medicine Keimyung University Daegu Republic of Korea; ^2^ Department of Chemistry College of Natural Sciences Keimyung University Daegu Republic of Korea; ^3^ Department of Physiology College of Medicine Keimyung University Daegu Republic of Korea; ^4^ Comparative Biomedical Sciences Royal Veterinary College London UK; ^5^ Faculty of Medicine Department of Pathology University of Tsukuba Tsukuba Japan; ^6^ Biological Disaster Analysis Group Division of Convergence Biotechnology Korea Basic Science Institute Daejeon Republic of Korea; ^7^ Graduate School of Analytical Science and Technology Chungnam National University Daejeon Republic of Korea

**Keywords:** amiloride, DKK‐3, macropinocytosis, meridianin C, YD‐10B

## Abstract

Meridianin C is a marine natural product known for its anti‐cancer activity. At present, the anti‐tumour effects of meridianin C on oral squamous cell carcinoma are unknown. Here, we investigated the effect of meridianin C on the proliferation of four different human tongue cancer cells, YD‐8, YD‐10B, YD‐38 and HSC‐3. Among the cells tested, meridianin C most strongly reduced the growth of YD‐10B cells; the most aggressive and tumorigenic of the cell lines tested. Strikingly, meridianin C induced a significant accumulation of macropinosomes in the YD‐10B cells; confirmed by the microscopic and TEM analysis as well as the entry of FITC‐dextran, which was sensitive to the macropinocytosis inhibitor amiloride. SEM data also revealed abundant long and thin membrane extensions that resemble lamellipodia on the surface of YD‐10B cells treated with meridianin C, pointing out that meridianin C‐induced macropinosomes was the result of macropinocytosis. In addition, meridianin C reduced cellular levels of Dickkopf‐related protein‐3 (DKK‐3), a known negative regulator of macropinocytosis. A role for DKK‐3 in regulating macropinocytosis in the YD‐10B cells was confirmed by siRNA knockdown of endogenous DKK‐3, which led to a partial accumulation of vacuoles and a reduction in cell proliferation, and by exogenous DKK‐3 overexpression, which resulted in a considerable inhibition of the meridianin C‐induced vacuole formation and decrease in cell survival. In summary, this is the first study reporting meridianin C has novel anti‐proliferative effects via macropinocytosis in the highly tumorigenic YD‐10B cell line and the effects are mediated in part through down‐regulation of DKK‐3.

## 1 INTRODUCTION

Oral squamous cell carcinoma (OSCC) is the most common malignant tumour of the lip, oral cavity and oropharynx. Moreover, OSCC can be severely disfiguring leading to a poor quality of life, including loss of general cognitive, social, emotional or physical functions as well as social relationships.^1^ The primary treatment of OSCC is surgical removal, but radiation therapy and/or chemotherapy have emerged as alternative treatment options for OSCC.^2^, ^3^, ^4^ Characteristically, OSCC cells at the late stage of malignancies are very resistant to cancer therapy‐mediated apoptosis. Thus, there is an urgent need for understanding resistance mechanism and identifying new drugs or substances which offer therapeutic efficacy against OSCC.

Macropinocytosis is an endocytic pathway that leads to the formation of single membrane irregular vesicles (macropinosomes) by the internalization of large patches of the plasma membrane along with associated extracellular fluid and solutes.^5^ Unlike the canonical and endocytic pathways that depend on coat proteins such as clathrin or caveolin, one of the distinctive hallmarks of macropinocytosis is the formation of expansive membrane ruffles within the plasma membrane.^6^ This membrane ruffling is initiated by the rapid polymerization of branching of actin filaments.^7^ Multiple proteins and signalling factors are involved in macropinocytosis, including the Rho superfamily of GTPases (Rac, Cdc42), lipid components (phosphoinositides, cholesterol, phosphatidylinositol phosphates), phospholipid kinases, receptor tyrosine kinases and phosphatases, SNX‐5 and DKK‐3.^8^, ^9^, ^10^, ^11^, ^12^, ^13^, ^14^, ^15^, ^16^ There is also large body of evidence that now shows macropinocytosis can be specifically activated by extracellular stimuli, such as growth factors and natural or synthetic chemicals, and the activated macropinocytosis can have effects on cell survival/proliferation or cell death/growth inhibition on distinct cell types including cancer cells.^17^, ^18^

Meridianin C is one of the marine derived indole alkaloids (meridianin A‐G) isolated from the South Atlantic tunicate *Aplidium meridianum*.^19^, ^20^ Previously, meridianin C, D or G analogues/derivatives have been shown to inhibit the proliferation of human breast (MCF‐7) and cervix (HeLa) cancer and leukaemia (MV4‐11) cells.^21^, ^22^, ^23^ At present, neither the anti‐tumour effect nor the mode of action of meridianin C in OSCC is known. In this study, we investigated the effect of meridianin C at 1 μM concentration on growth of four different human oral carcinoma cell lines (YD‐8, YD‐10B, YD‐38 and HSC‐3) that were originated from tongue (YD‐8, YD‐10B, HSC‐3) or lower gingiva (YD‐38).^24^ It is worth noting that YD‐8 and YD‐38 cells have no tumorigenicity, but YD‐10B cells are highly tumorigenic and invasive/metastatic.^25^, ^26^ In this article, we report for the first time that meridianin C strongly inhibits the growth of YD‐10B OSCC cells through macropinocytosis, and its growth inhibitory and macropinocytosis inducing effects on the cells are mediated in part via the reduced expression of DKK‐3.

## 2 MATERIAL AND METHODS

### 2.1 Cell culture and reagents

Four different human tongue cancer cell lines (YD‐8, YD‐10B, YD‐38, HSC‐3) and a human bladder cancer cell line (T24) were purchased from Korean Cell Line Bank (Seoul, Republic of Korea). Normal human gingival fibroblasts (HGF) were obtained from American Type Culture Collection (ATCC, CRL‐2014^™^, Manassas, VA).Cells were cultured in RPMI 1640 medium supplemented with 10% heat‐inactivated foetal bovine serum (FBS), 100 units/mL penicillin and 100 μg/mL streptomycin and maintained at 37°C in a humidified condition of 95% air and 5% CO_2_. Meridianin C was synthesized as previously described 23 and prepared as a 10 mM stock solution in DMSO. RPMI‐1640 (LM011‐01), FBS (S001‐01) and penicillin–streptomycin (LS202‐02) were purchased from Welgene (Deagu, Republic of Korea). All commercial antibodies and chemicals were purchased from the following resources: anti‐procaspase‐9 (ADI‐AAM‐139) was from Enzo (Farmingdale, NY, USA); anti‐PARP (11835238001) was from Roche Diagnostics (Mannheim, Germany); anti‐DR5 (NBP1‐45951) was from Novus Biologicals (Littleton, CO, USA); anti‐actin (A5441) was from Sigma (St. Louis, MO, USA); anti‐DKK‐3 (ab187532) was from Abcam (Cambridge, MA, USA); anti‐Flag (#2368) from Cell Signalling Technology (Danvers, MA, USA); FITC‐dextran (average molecular weight of 70 kDa) (46945) was from Sigma (St. Louis, MO, USA); z‐VAD‐fmk (627610) was from Calbiochem (Madison, WI, USA); Amiloride (A7410) was from Sigma (St. Louis, MO, USA) and protease inhibitor cocktail (PIC) (100x) (539134) were from Calbiochem (Madison, WI, USA). SuperSignal^™^ West Pico PLUS Enhanced chemiluminescence (ECL, #34080) was purchased from Thermo Scientific (Waltham, MA, USA). Plasticware: 6‐well plates, 24‐well plates and 60 or 100 mm cell culture dish were purchased from SPL Life Sciences (Gyeonggi‐do, Korea). Control siRNA (sc‐37007) and DKK‐3 siRNA (sc‐41102) were purchased from Santa Cruz biotechnology (Delaware, CA, USA).

### 2.2 Cell count and cell morphology analysis

Respective OSCC cell line (YD‐8, YD‐10B, YD‐38 or HSC‐3) was seeded in 24‐well plates (1 × 10^5^/500 μL/well) overnight. After cells were treated with meridianin C (1 μM) or vehicle control (DMSO; 0.1%) for 48 h, surviving cells, which cannot be stained with trypan blue dye, were counted using phase contrast microscope. Approximately 100 cells were counted for each evaluation. The cell count assay was performed in triplicate. Data are mean ± SEM of three independent experiments. Survival is expressed as a percentage of control. In some experiments, YD‐10B cells (0.5 × 10^6^/2 mL/well) or normal HGFs‐1) (0.4 × 10^6^/2 mL/well) were also plated in 6‐well plates overnight. Cells were then treated with meridianin C (1 μM) or DMSO for indicated times (YD‐10B cells for 2, 4, 8 or 24 h; normal HGFs for 4, 8, 24 or 48 h). Phase contrast images of the conditioned cells were taken with an Olympus phase contrast microscope equipped with a digital camera (Nikon).

### 2.3 Transmission electron microscopy (TEM)

YD‐10B cells (0.5 × 10^6^/2 mL/well) were seeded in 6‐well plates the day before meridianin C treatment. Cells were then treated with 1 μM meridianin C for 8 h, while DMSO was used as control. DMSO or meridianin C‐treated cells were fixed overnight at 4°C with 2.5% glutaraldehyde in 0.1 M sodium cacodylate buffer, pH 7.4. Samples were post‐fixed for 30 min with 0.5% osmium tetroxide/0.8% potassium ferricyanide, transferred to 1% tannic acid for 1 h and then to 1% uranyl acetate overnight at 4°C. Next, the samples were dehydrated with a graded ethanol series and embedded in Spurr's resin. Thin sections were cut with a Leica EM UC6 ultramicrotome (Leica), and stained with 1% uranyl acetate and Reynold's lead citrate prior to viewing at 120 kV on a Tecnai BT Spirit transmission electron microscope (FEI). Digital images were acquired with a Hammamatsu XR‐100 side mount digital camera system (Advanced Microscopy Techniques) and processed using Adobe Photoshop CS5 (Adobe Systems Inc.).

### 2.4 Scanning electron microscopy

YD‐10B cells were seeded in 6‐well plates (0.5 × 10^6^ cells/2 mL/well) the day before meridianin C treatment. Cells were then treated with vehicle control (DMSO) or 1 μM meridianin C for 2 h. The conditioned cells were centrifuged, fixed in 0.5% glutaraldehyde and 0.5% paraformaldehyde fixative, washed with 0.1 M phosphate‐buffered saline (PBS), and post‐fixed with 1% osmium tetroxide solution for 1 h. 2% tannic acid was used to conductively stain for 12 h, later washed with PBS, and fixed with 1% osmium tetroxide solution for 1 h. The specimens were dehydrated with ethanol, *t*‐butyl alcohol and freeze dryer. Finally, the specimen was sputter coated with platinum‐palladium (Pt‐Pd) in an ion coater for 2 min, followed by microscopic examinations (S‐4200, Hitachi Co., Tokyo, Japan).

### 2.5 Measurement of DNA fragmentation

YD‐10B cells were seeded in 6‐well plates at a density of 0.5 × 10^6^ cells per well the day before treatment. Cells were incubated with meridianin C or vehicle control (DMSO) for 4 to 48 h, at which point, cells were harvested, washed and lysed in a buffer containing 50 mM Tris (pH 8.0), 0.5% sarkosyl, 0.5 mg/mL proteinase K and 1 mM EDTA at 55°C for 3 h, followed by addition of RNase A (0.5 μg/mL) for a further 18 h at 55°C. The lysates were then centrifuged at 10 000 *g* for 20 min, genomic DNA in the supernatant was extracted with equal volume of neutral phenol–chloroform–isoamyl alcohol mixture (25:24:1), and analysed by electrophoresis on a 1.7% agarose gel. The DNA was visualized and photographed under UV illumination after staining with ethidium bromide (0.1 μg/mL).

### 2.6 Measurement of the population of sub G1 phase by flow cytometry analysis

After 24‐ or 48‐h treatment with DMSO or meridianin C (1 μM), YD‐10 B cells were harvested and washed with PBS, fixed in ice‐cold 70% ethanol and stored at 4°C. Cells were then washed once with PBS, suspended in 1 mL of cold propidium iodide (PI) solution containing 100 μg/mL RNase A, 50 μg/mL propidium iodide, 0.1% (w/v) sodium citrate and 0.1% (v/v) NP‐40 and incubated on ice for additional 30 min in the darkness. Cytometric analyses were carried out with a flow cytometer (FACS Caliber, Becton Dikinson) and CellQuest software. Approximately, 10 000 cells were counted for the analysis.

### 2.7 Fluorescein isothiocyanate (FITC) staining

To monitor the functionality of meridianin C‐induced macropinocytosis (macropinosome formation/internalization), 0.25 × 10^5^ YD‐10B cells/mL were seeded on coverslips and treated with meridianin C (1 μM) and/or FITC‐dextran (0.5 mg/mL) in the presence or absence of amiloride (4 mM) for 4 h. The cells were washed twice with PBS and mounted onto microscopic glass slides using Permafluor aqueous mounting media (Thermo Scientific, Waltham, MA, USA) media. Bright field and fluorescence were observed using a Zeiss AxioObserver.A1 inverted microscope (Carl Zeiss, Germany) and images acquired using Zen 2 software (Carl Zeiss). Fluorescent intensity was quantified using Image‐J software.

### 2.8 Preparation of whole cell lysates

To see the effect of meridianin C on expression of apoptosis‐ or macropinocytosis‐related proteins, YD‐10B cells (0.5 × 10^6^/2 mL/well) were seeded in 6‐well plates the day before meridianin C treatment. Cells were treated with meridianin C (1 μM) or vehicle control (DMSO) for the indicated times. At each time‐point, cells were washed twice with PBS and proteins extracted using a modified RIPA buffer (50 mM Tris‐Cl (pH 7.4), 150 mM NaCl, 0.1% sodium dodecyl sulphate, 0.25% sodium deoxycholate, 1% Triton X‐100, 1% Nonidet P‐40, 1 mM EDTA, 1 mM EGTA, PIC (1×)). The cell lysates were collected and centrifuged at 12 000 rpm for 20 min at 4°C. The supernatants were saved and protein concentrations determined by bicinchoninic acid assay (BCA) protein assay (Pierce).

### 2.9 Immunoblot analysis

Proteins (50 μg) were separated by SDS‐PAGE (10%) and transferred onto nitrocellulose membranes (Millipore, Bedford, MA, USA). The membranes were washed with TBS (10 mM Tris‐Cl, 150 mM NaCl, pH 7.5) with 0.05% (v/v) Tween‐20 followed by blocking with TBST containing 5% (w/v) non‐fat dried milk. The membranes were incubated overnight with antibodies specific for procaspase‐9 (1:1000), DR‐5 (1:1000), PARP (1:2000), DKK‐3 (1:1000), Flag (1:1000) or β‐actin (1:10 000) at 4°C. The membranes were then exposed to secondary antibodies conjugated to horseradish peroxidase for 2 h at room temperature and further washed three times with TBST. Immunoreactivity was detected by SuperSignal^™^ West Pico PLUS enhanced chemiluminescence (ECL) according to manufacturer (Thermo Scientific, Waltham, MA, USA). Equal protein loading was assessed by the expression levels of β‐actin.

### 2.10 Small interfering RNA (siRNA) transfection

YD‐10B cells (0.5 × 10^5^/well) seeded into 6‐well plates were transfected for 6 h with control or DKK‐3 siRNA (80 pM) using LipofectamineTM 2000 (Invitrogen, Carlsbad, CA, USA). Culture medium from the transfected cells was removed and refreshed with RPMI containing 10% FBS, followed by incubation for 18 h. The knockdown efficiency of the DKK‐3 siRNA was determined by immunoblotting in comparison to β‐actin.

### 2.11 Generation of stable YD‐10B cells overexpressing DKK‐3

The pcDNA3.1‐DKK3‐C‐Flag or mock vector was constructed as reported previously.^27^ Briefly, YD‐10B cells were transfected in a stable manner with the pcDNA3.1‐DKK‐3‐Flag or mock vector using LipofectAMINE transfection reagent as prescribed by the manufacturer (Invitrogen, Carlsbad, CA, USA). After 48 h of incubation, transfected cells were selected in primary cell culture medium containing 200 μg/mL G418 (Promega, Madison, WI, USA). After 2 or 3 weeks, to rule out the possibility of clonal differences between the generated stable cell lines, the pooled YD‐10B/mock vector and YD‐10B/DKK‐3‐Flag clones were tested for DKK‐3 and Flag expression by immunoblotting and were used in this study.

### 2.12 Statistical analyses

Cell count analysis was performed in triplicates and repeated three times. Data were expressed as mean ± SEM. The significance of difference was determined by one‐way ANOVA (Laerd Statistics, Chicago, IL, USA). All significance testing was based upon a *P* < 0.05.

## 3 RESULTS

### 3.1 Meridianin C has strong growth inhibitory and vacuole‐inducing effects on YD‐10B cells

**Figure 1:**
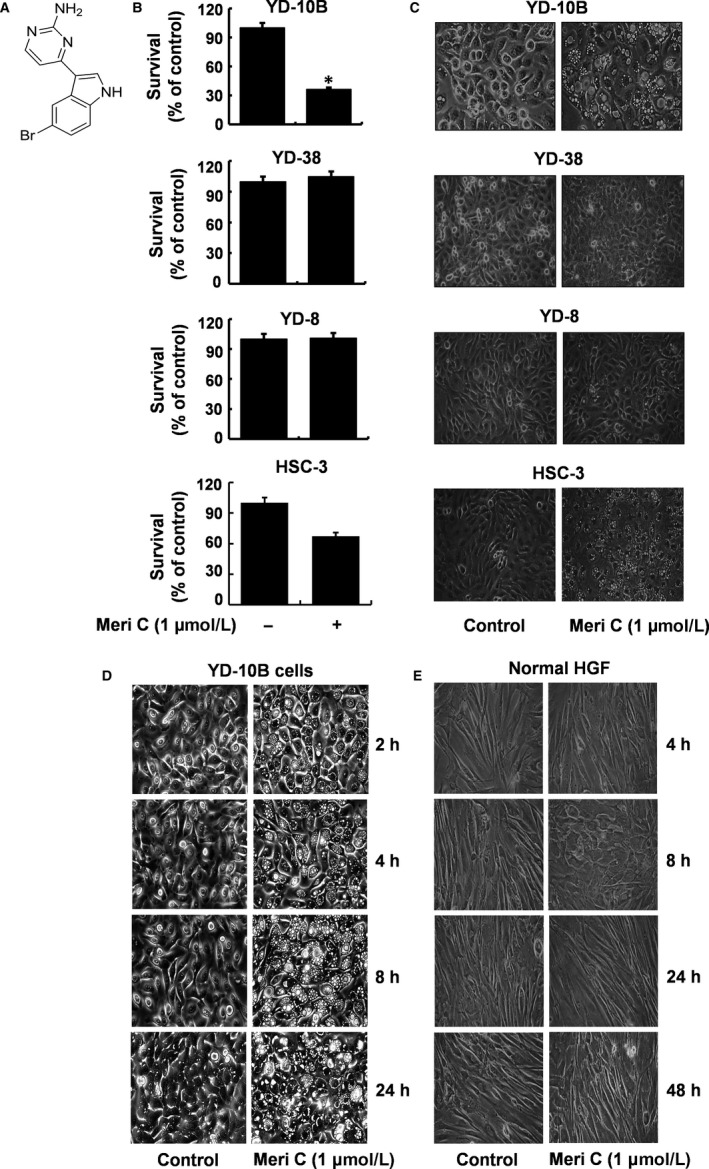
Meridianin C inhibits YD‐10B cell survival and induces cellular vacuole formation. (A) The chemical structure of meridianin C. (B) Four human tongue cancer cell lines (YD‐8, YD‐10B,YD‐38, and HSC‐3), respectively, were treated with meridianin C (1 μM) or vehicle control (DMSO; 0.1%) for 48 h. The number of surviving cells was measured by trypan blue dye exclusion. Data are mean ± SEM of three independent experiments, each done in triplicate. *P < 0.05 compared to the value of meridianin C free control at the indicated time. (C) YD‐8, YD‐10B, YD‐38 and HSC‐3 cells, respectively, were treated with meridianin C (1 μM) or vehicle control (DMSO) for 48 h. Images of the conditioned cells were obtained by phase contrast microscopy, 400 X. Each image is a representative of three independent experiments. (D, E) YD‐10B cells or normal human gingival fibroblasts (HGF) were treated with meridianin C (1 μM) or vehicle control (DMSO) for the times indicated. At each time‐point, images were obtained by phase contrast microscopy, 400 X. Each image is a representative of three independent experiments

We investigated the treatment effect of meridianin C (Figure 1A) at 1 μM concentration for 48 h on the growth of four different human tongue carcinoma cell lines (YD‐8, YD‐10B, YD‐38 and HSC‐3) by cell count analysis. Among the cell lines tested, YD‐10B cells were the most sensitive to meridianin C (Figure 1B). Meridianin C treatment led to a slight reduction in HSC‐3 cells, which was statically insignificant. Both YD‐38 and YD‐8 and cells were insensitive to meridianin C treatment. Strikingly, YD‐10B cells treated with meridianin C were highly vacuolated (Figure 1C). Furthermore, HSC‐3 cells treated with meridianin C also formed many vacuoles in much smaller size than those in YD‐10B cells. However, both YD‐38 and YD‐8 cells treated with meridianin C had very low or no cytoplasmic vacuoles. Meridianin C caused a time‐dependent increase in cytoplasmic vacuole formation in YD‐10B cells, with effects visible by 2 h (Figure 1D). We next examined the effect of meridianin C on growth and accumulation of vacuoles in normal human gingival fibroblast (HGF), but no cytoplasmic vacuoles were observed at 4, 8, 24 or 48 h, and meridianin C was not cytotoxic to HGF (Figure 1E). However, it was shown that treatment with meridianin C at higher concentrations (5 or 10 μM) and different times (2, 4, 8, 24 or 48 h) strongly reduced survival and induced massive vacuoles of both YD‐10B and normal HGFs, pointing out that meridianin C at higher concentrations (5 or 10 μM) than 1 μM are cytotoxic to YD‐10B cells as well as normal HGFs. These results show that meridianin C has a strong and selective anti‐survival and vacuole‐inducing effects on the highly invasive and tumorigenic YD‐10B cells at the 1 μM concentration. Therefore, we decided to focus on the YD‐10B cell line and the 1 μM concentration of meridianin C for further studies.

### 3.2 Meridianin C does not induce apoptosis in YD‐10B cells

**Figure 2:**
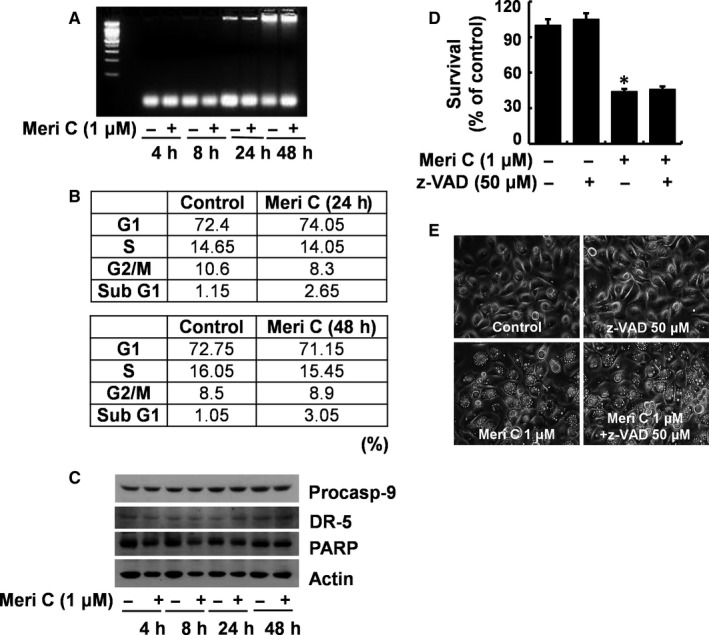
Meridianin C does not induce apoptosis of YD‐10B cells. (A) YD‐10B cells were treated with meridianin C (1 μM) or vehicle control (DMSO) for the times indicated. At each time‐point, extra‐nuclear fragmented DNA was extracted, and analysed on a 1.7% agarose gel. The images are representative of three independent experiments. SM, DNA size marker. (B) YD‐10B cells were treated with meridianin C (1 μM) or vehicle control (DMSO) for the times indicated and evaluated for apoptotic subG1 DNA content by flow cytometer. The tables represent the fraction of apoptotic cells. Data are mean ± SEM of three independent experiments. (C) YD‐10B cells were treated with meridianin C (1 μM) or vehicle control (DMSO) for the times designated. At each time‐point, whole cell lysates were prepared, and analysed for procaspase‐9, DR5, PARP cleavage and β‐actin by Western blotting. The images are representative of three independent experiments. (D) YD‐10B cells were treated with meridianin C (1 μM) in the absence or presence of z‐VAD (50 μM), a pan‐caspase inhibitor, for 48 h, and cell survival counted by the trypan blue dye exclusion assay. Data are mean ± SEM of three independent experiments, each performed in triplicate. *P < 0.05 compared to the control at the indicated time. (E) YD‐10B cells were treated with meridianin C (1 μM) in the absence or presence of z‐VAD (50 μM), a pan‐caspase inhibitor, for 48 h. Images were obtained by phase contrast microscopy, 400 X. Each image is representative of three independent experiments

We next determined whether meridianin C treatment induces apoptosis in YD‐10B cells by nuclear DNA fragmentation assay. As shown in Figure 2A, no apoptotic DNA fragmentation occurred in YD‐10B cells when treated with meridianin C for 4, 8, 24 or 48 h. Further to confirm apoptosis, we analysed the amount of subG1 DNA by flow cytometry in YD‐10B cells treated with 1 μM meridianin C for the indicated times (24, 48 h). As shown in Figure 2B, the addition of meridianin C did not result in increased accumulation of sub‐G1 phase, a well‐known apoptotic marker. Further, Western blotting analysis revealed that meridianin C treatment did not affect expression levels of apoptosis‐related proteins, procaspase‐9 and death receptor‐5 (DR‐5), or PARP cleavage (Figure 2C) until 48 h of treatment. Moreover, treatment with z‐VAD‐fmk, a pan‐caspase inhibitor 28 did not block the meridianin C‐induced growth inhibition and accumulation of vacuoles in YD‐10B cells (Figure 2D, E).

### 3.3 Meridianin C induces macropinocytosis (macropinosomes) in YD‐10B cells

**Figure 3:**
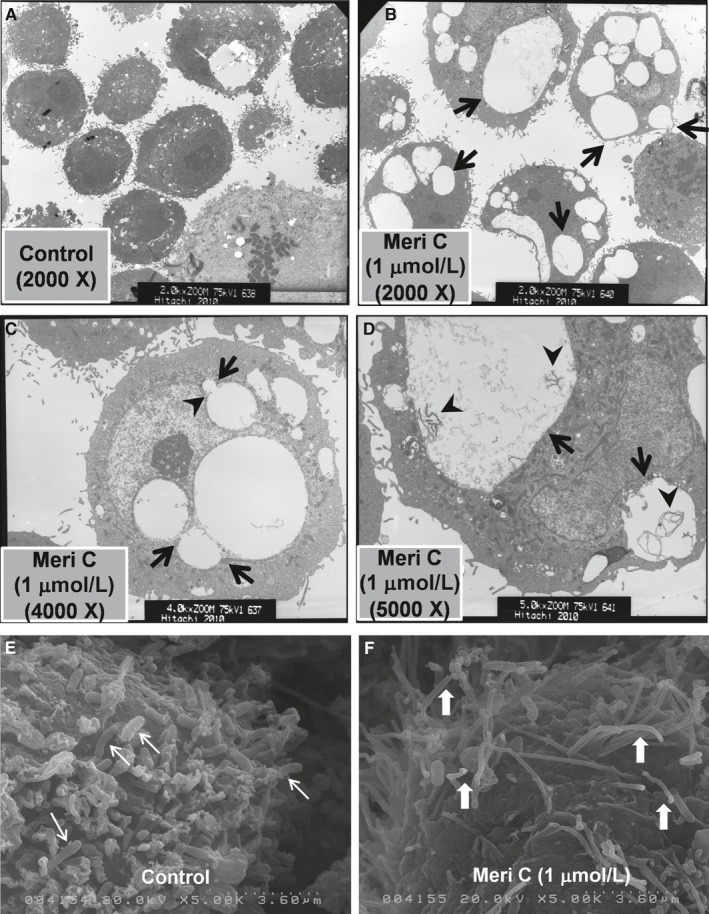
Transmission and scanning electron micrographs of meridianin C‐induced macropinosomes and macropinocytosis in YD‐10B cells. (A‐D) YD‐10B cells were treated with meridianin C (1 μM) or vehicle control (DMSO) for 8 h. (A) Vehicle (DMSO)‐treated cells, 2000 X. (B) Meridianin C‐treated cells, 2000 X. Arrows indicate the cytoplasmic vacuoles of different sizes. (C) Meridianin C‐treated cells, 4000 X. Arrowhead indicates the indented or distorted nucleus because of enlarged vacuole. Arrows indicate fusion between cytoplasmic vacuoles of different sizes. (D) Meridianin C‐treated cells, 5000 X. Arrows indicate macropinosomes containing membranous contents in different sizes, which are shown by arrowheads. (E, F) YD‐10B cells were treated with meridianin C (1 μM) or vehicle control (DMSO) for 2 h. (E) Vehicle control treated cells, which show many short and thick bacilli bound to the cell surface (thin arrows). (F) Meridianin C‐treated cells, which exhibit abundant long and thin membrane extensions that resemble lamellipodia on the cells surface (wide white arrows)

Using transmission electron microscopy (TEM), we investigated whether vacuoles induced by meridianin C in YD‐10B cells are macropinosomes. While YD‐10B cells treated with vehicle control (DMSO) had no or tiny vacuoles in the cytoplasm (Figure 3A), the meridianin C‐treated cells displayed ultrastructural characteristics of macropinosomes (vacuoles) in different sizes (arrows) (Figure 3B). Under high magnification, the meridianin C‐treated YD‐10B cells showed fusion between large and small vacuoles in the cytoplasm (arrows) and indentation of the nuclear membrane (arrowhead) probably because of enlarged vacuolar structure (Figure 3C). Furthermore, residual materials, including membranous debris (arrowheads), were seen within the cytoplasmic vacuoles (arrows) in YD‐10B cells treated with meridianin C (Figure 3D). It is documented that macropinosomes are derived from membrane ruffles and protrusions folding back and fusing with the plasma membrane to form large vesicles.^29^ Further, to confirm that the formation of macropinosomes induced by meridianin C is the result of macropinocytosis, scanning electron microscopy (SEM) was used to see the effect of meridianin C on induction of membrane ruffling or lamellipodia formation in YD‐10B cells. Apparently, while vehicle control (DMSO)‐treated YD‐10B cells had many short and thick bacilli bound to the cell surface (thin arrows) (Figure 3E), there were abundant long and thin membrane extensions that resemble lamellipodia (wide white arrows) on the surface of cells treated with meridianin C for 2 h (Figure 3F).

### 3.4 Meridianin C‐induced macropinocytosis (macropinosome formation/internalization) in YD‐10B cells is functional

**Figure 4:**
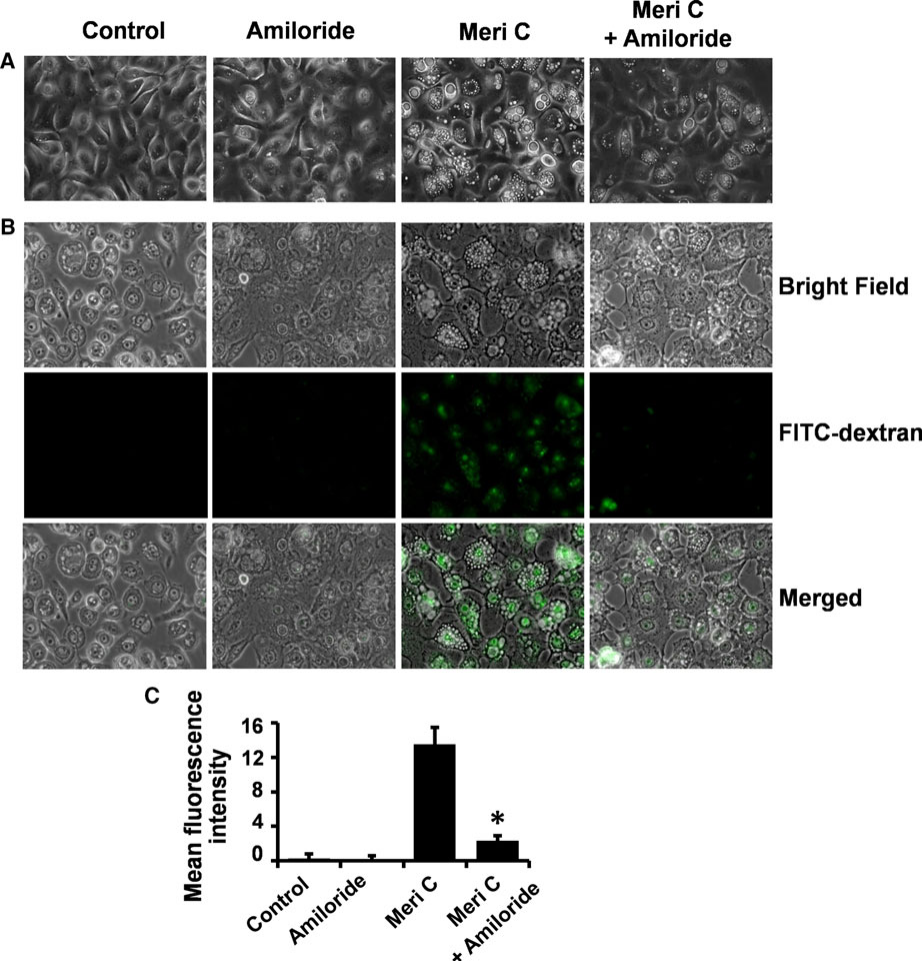
Suppression of meridianin C‐induced macropinocytosis (macropinosomes) in YD‐10B cells by amiloride, a macropinocytosis inhibitor. (A) YD‐10B cells were treated with meridianin C (1 μM) in the absence or presence of amiloride (4 mM) for 4 h. Images were obtained by phase contrast microscopy, 400 X. Each image is a representative of three independent experiments. (B) YD‐10B cells were seeded on coverslips and treated with meridianin C (1 μM) and/or FITC‐dextran (0.5 mg/ml) in the absence or presence of amiloride (4 mM) for 4 h. The cells were washed and mounted onto microscopic glass slides. Bright field and fluorescence were observed with a Zeiss AxioObserver.A1 inverted microscope and images were acquired using Zen 2 software. (C) Fluorescence intensity of FITC‐dextran (green colour) was determined by Image‐J software. Each value in the graph represents the mean ± SEM of three independent experiments. *P < 0.05 compared to the meridian C treatment at the indicated time

Amiloride inhibits macropinocytosis by blocking the activity of Na/H exchanger, causing a decrease in pH at the plasma membrane.^30^ This amiloride‐induced pH drop suppresses the membrane ruffling and membrane extensions that form the macropinosome.^31^ Soluble sugars or proteins, such as dextran or albumin, are useful fluorescent markers specific for macropinocytosis.^32^ Using amiloride and fluorescein isothiocyanate‐dextran with molecular weight of 70 kDa (FITC‐dextran), we sought to explore whether the meridianin C‐induced macropinocytosis (macropinosome formation/internalization) in YD‐10B cells is functional. Microscopic observations clearly demonstrated that amiloride strongly blocked the meridianin C‐induced accumulation of vacuoles in YD‐10B cells (Figure 4A). Moreover, meridianin C increased the delivery of FITC‐dextran into YD‐10B cells while amiloride strongly inhibited it (Figure 4B). Further, fluorescence intensity of FITC‐dextran (green colour) was determined by Image‐J software (Figure 4C).

### 3.5 Meridianin C‐induced down‐regulation of DKK‐3 protein in YD‐10B cells

**Figure 5:**
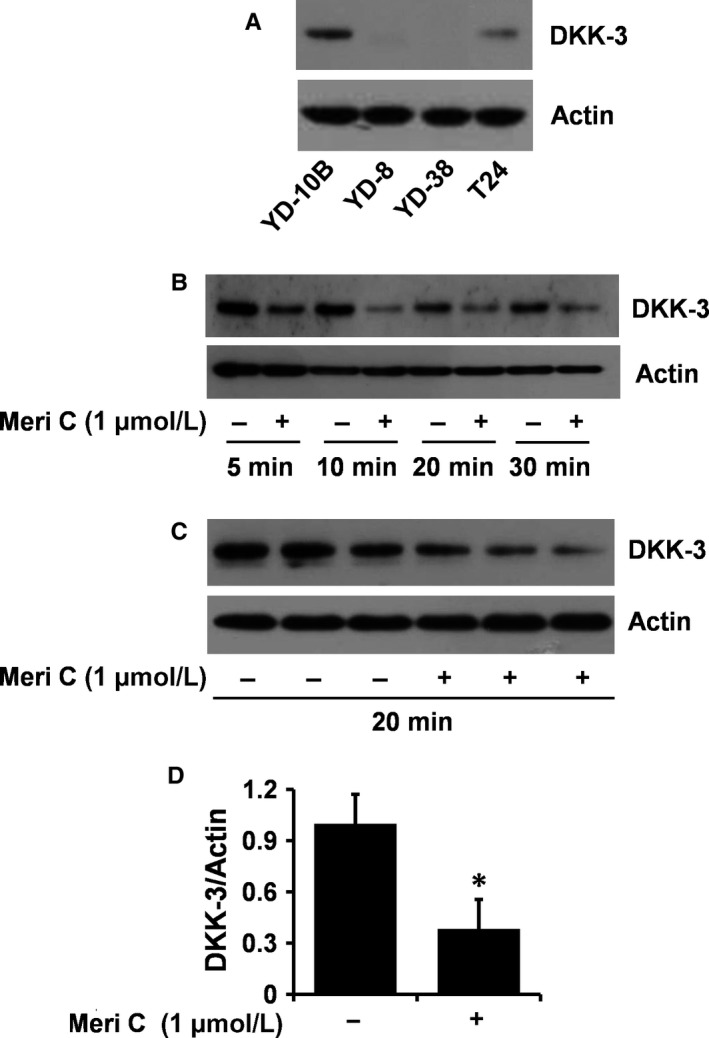
Effect of meridianin C on expression of DKK‐3 in YD‐10B cells. (A) Whole cell lysates of YD‐8, YD‐10B, YD‐38 or T24 cells were analysed for DKK‐3 or β‐actin by Western blotting. (B) YD‐10B cells were treated with meridianin C (1 μM) or vehicle control (DMSO) for the times designated or (C) for 20 min. At each time‐point, whole cell lysates were prepared, and analysed for DKK‐3 compared to β‐actin by Western blotting. (D) The densitometry data of (C). *P < 0.05 compared to the control (−) at the indicated time

DKK‐3 plays a role in modulating macropinocytosis in T24 human bladder cancer cells 16 and is also expressed in OSCC‐derived cell lines.^33^ We therefore sought to explore whether DKK‐3 is expressed in YD‐10B cells and is regulated by meridianin C treatment. As shown in Figure 5A, high cellular levels of DKK‐3 protein were seen in YD‐10B cells, and its expression level was even higher relative to β‐actin than that in T24 cells. There was no detectable DKK‐3 protein expression in YD‐8 or YD‐38 cells. Time course experiments further revealed that meridianin C treatment led to a rapid time‐dependent reduction in DKK‐3 protein in YD‐10B cells (Figure 5B‐D).

### 3.6 DKK‐3 knock‐down led to partial accumulation of vacuoles in YD‐10B cells along with partial decrease of the cell growth

**Figure 6:**
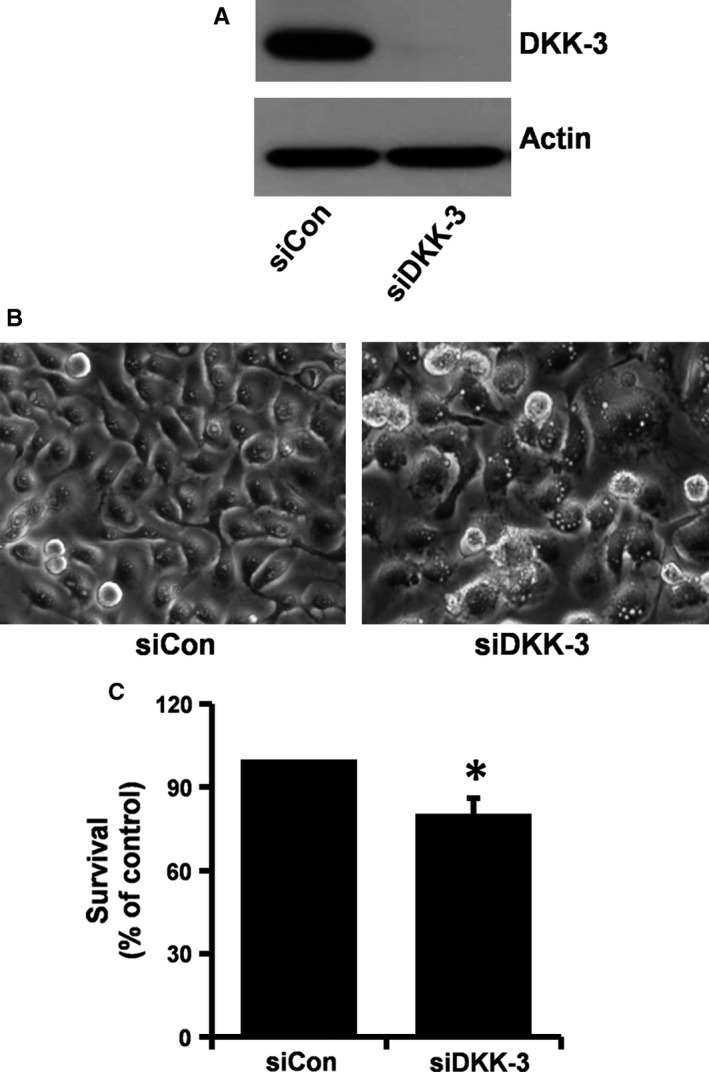
Effects of DKK‐3 knockdown on accumulation of vacuoles and growth of YD‐10B cells. (A) YD‐10B cells were transfected with 80 pM of control or DKK‐3 siRNA for 24 h. Whole cell lysates were prepared, and analysed by Western blot. The pictures are representative of three independent experiments. (B) YD‐10B cells were transfected with 80 pM of control or DKK‐3 siRNA for 24 h. Images were obtained by phase contrast microscopy, 400 X. Each image is representative of three independent experiments. (C) YD‐10B cells were transfected with 80 pM of control or DKK‐3 siRNA for 24 h, and cell survival counted by the trypan blue dye exclusion assay. Data are mean ± SEM of three independent experiments, each performed in triplicate. *P < 0.05 compared to the value of control siRNA at the indicated time

To evaluate the role of reduced DKK‐3 in meridianin C's growth inhibition and/or macropinocytosis in YD‐10B cells, we transfected control or DKK‐3 siRNA into YD‐10B cells and measured the number of surviving cells and accumulation of cytoplasmic vacuoles. There was almost complete knock‐down of DKK‐3 in the DKK‐3 siRNA‐transfected YD‐10B cells, compared with the control siRNA‐transfected ones (Figure 6A), suggesting high DKK‐3 siRNA transfection efficiency. YD‐10B cells transfected with DKK‐3 siRNA had partial accumulation of vacuoles (Figure 6B) accompanied with a partial but significant reduction in cell survival (Figure 6C).

### 3.7 Overexpression of DKK‐3 largely blocks meridianin C‐induced accumulation of vacuoles in YD‐10B cells along with significant increase of the cell survival

**Figure 7:**
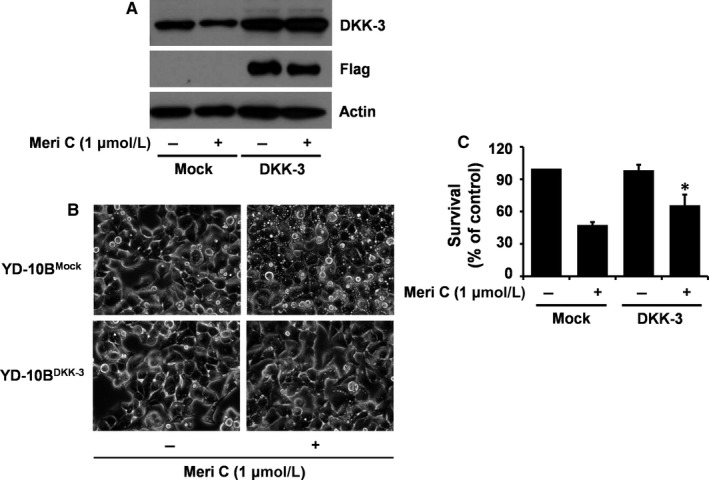
Effects of DKK‐3 overexpression on meridianin C‐induced accumulation of vacuoles and decrease in survival of YD‐10B cells. (A) YD‐10B cells were transfected with pcDNA3.1‐DKK‐3‐Flag or mock vector using LipofectAMINE 2000 reagents. Stable clones were selected in the culture media containing 200 μg/ml G418 sulphate for several weeks. The selected clones (DKK‐3‐Flag or mock vector‐transfected YD‐10B cells) were seeded overnight. Cells were then treated with or without meridianin C (1 μM) for 20 min. Cell lysates were prepared and analysed by Western blotting for measurement of the expression levels of DKK‐3, Flag and β‐actin. (B) DKK‐3‐ or mock‐transfected YD‐10B cells were treated vehicle or meridianin C (1 μM) for 4 h. Images were obtained by phase contrast microscopy, 400 X. Each image is a representative of three independent experiments. (C) DKK‐3‐ or mock‐transfected YD‐10B cells were treated with or without meridianin C (1 μM) for 24 h, followed measurement of the number of cells survived by the trypan blue dye exclusion assay. Data are mean ± SEM of three independent experiments, each performed in triplicate. *P < 0.05 compared to the control at the indicated time

To further clarify that DKK‐3 plays an important role in the function of meridianin C, we next performed DKK‐3‐Flag cDNA transfection experiments to generate stable YD‐10B cells that overexpress DKK‐3‐Flag. Using these stable clones, we tested whether DKK‐3 overexpression could interfere with the meridianin C's growth inhibitory and vacuole‐inducing activities in YD‐10B cells. As shown in Figure 7A, there was a large increase in DKK‐3 protein levels in YD‐10B^DKK‐3^ cells (cells overexpressing DKK‐3‐Flag) compared with YD‐10B^mock^ cells (cells transfected with mock vector). High levels of Flag expression in YD‐10B^DKK‐3^, but not in YD‐10B^mock^, cells further support the DKK‐3 cDNA transfection efficiency. As expected, meridianin C led to a decrease in DKK‐3 protein levels in YD‐10B^mock^ cells. However, meridianin C did not influence DKK‐3 protein expression in YD‐10B^DKK‐3^ cells, although it slightly reduced Flag levels in the cells. Importantly, while meridianin C treatment largely induced many vacuoles in YD‐10B^mock^ cells, it had no effect on the formation of vacuoles in YD‐10B^DKK‐3^ cells (Figure 7B). Furthermore, as shown in Figure 7C, the meridianin C‐induced reduction in YD‐10B cell survival was considerably blocked by DKK‐3 overexpression.

### 3.8 The meridianin C's growth inhibitory and macropinocytosis inducing effects on YD‐10B cells are reversible

**Figure 8:**
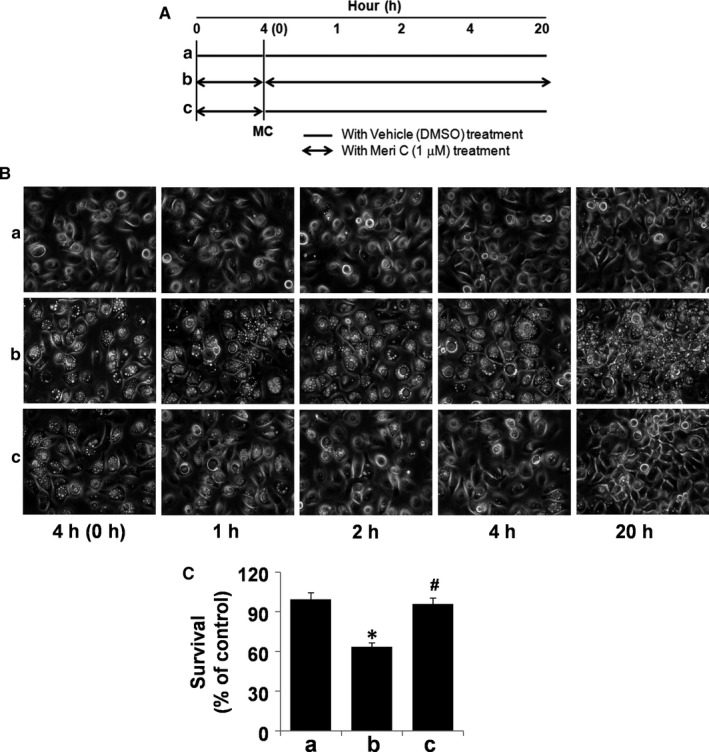
Reversibility of the meridianin C's actions on macropinocytosis and cell survival in YD‐10B cells. (A) Schematic outline of the experimental setup: The double‐headed arrows indicate the length of meridianin C (1 μM) treatment. (B) YD‐10B cells were treated with meridianin C (1 μM) or vehicle control (DMSO) for the indicated time periods. At each time‐point, images were obtained by phase contrast microscopy, 400 X. Each image is representative of three independent experiments. (C) YD‐10B cells were treated with meridianin C (1 μM) or vehicle control (DMSO) for the indicated time periods, and the cell count assay performed in triplicate. Data are mean ± SEM of three independent experiments. *P < 0.05 compared to the control at the indicated time. #*P* < 0.05 compared to the value of meridianin C at the indicated time‐point

To determine whether meridianin C exerts its effects on YD‐10B cell survival and macropinocytosis in a reversible or irreversible manner, YD‐10B cells were incubated with meridianin C or DMSO solvent control at the designated time‐points, as illustrated in Figure 8A. YD‐10B cells that were incubated with vehicle DMSO control for 4 h and then incubated with DMSO vehicle for an additional 1, 2, 4 and 20 h, showed no vacuole formation (treatment **a**) (Figure 8B). In contrast, YD‐10B cells that were incubated with meridianin C for 4 h and then further incubated with meridianin C for an additional 1, 2, 4 and 20 h, respectively, showed many vacuoles (treatment **b**). However, YD‐10B cells that were incubated with meridianin C for 4 h and then incubated with DMSO vehicle control for an additional 1, 2, 4 and 20 h, respectively, showed a time‐dependent reduction in vacuole number (treatment **c**). Importantly, the survival of YD‐10B cells in treatment **c** was much higher than that of the cells in treatment **b** (Figure 8C). These results show that the growth inhibitory and macropinocytosis inducing effects of meridianin C on YD‐10B cells do occur in a reversible manner.

### 3.9 Comparison of the effects of meridianin C and its derivatives on growth and macropinosome (vacuole) accumulation in YD‐10B cells

**Figure 9:**
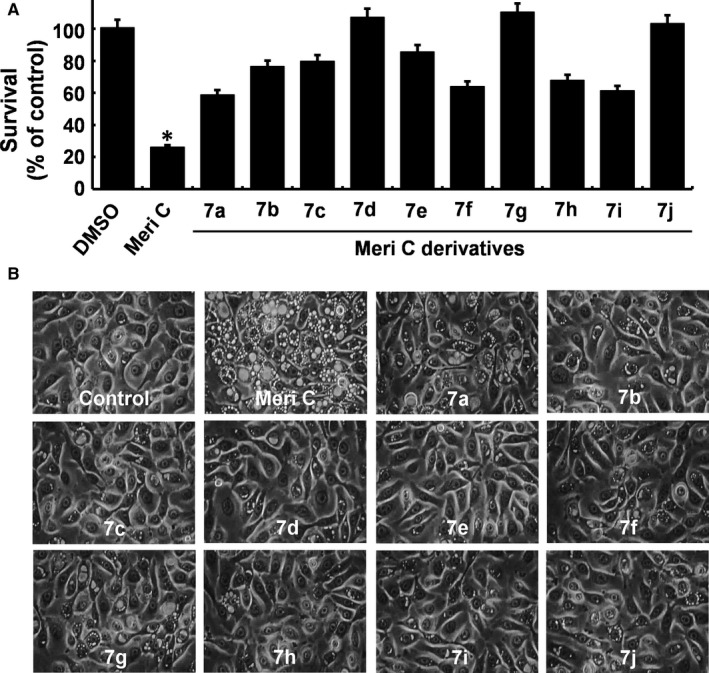
Effects of meridianin C and its derivatives on growth and vacuole formation of YD‐10B cells. (A) YD‐10B cells were treated with 1 μM of meridianin C or each derivative of meridianin C (a to j) for 48 h, and the cell count assay performed in triplicate. Data are mean ± SEM of three independent experiments. *P < 0.05 compared to the control at the indicated time. (B) YD‐10B cells were treated with 1 μM of meridianin C or each derivative (a to j) for 48 h. Images were obtained by phase contrast microscopy, 400 X. Each image is representative of three independent experiments

We have recently synthesized a novel series of meridianin C derivatives substituted at C‐5 position (7a‐j) and reported their anti‐proliferative activities against human leukaemic cell lines.^23^ In this study, we tested these meridianin C derivatives on growth and macropinosome (vacuole) accumulation of YD‐10B cells. Meridianin C treatment for 48 h strongly inhibited the survival of YD‐10B cells (Figure 9A). However, treatment with 7a, 7b, 7c, 7f, 7e, 7 h or 7i only slightly decreased of the cell survival. Treatment with 7d, 7 g or 7j had no effect on the survival of YD‐10B cells. Similarly, meridianin C treatment induced a significant accumulation of vacuoles in YD‐10B cells, whereas compounds 7a‐j had no or very low vacuole‐inducing activity (Figure 9B).

## 4 DISCUSSION

Macropinocytosis can be activated and exploited in normal and cancer cells to confer a cell growth/survival advantage.^34^, ^35^, ^36^ By contrast, hyper‐stimulation of macropinocytosis might play a critical role in the cytotoxicity of normal and cancer cells.^35^, ^37^ Notably, there are a number of several natural or synthetic chemicals that have anti‐tumour effects through stimulation of macropinocytosis.^38^, ^39^ Indeed, it has been suggested that any substance that specifically and massively induces macropinocytosis in cancer cells may have therapeutic potential as an anti‐tumour agent. Regulation of human tongue cancer cell growth and macropinocytosis by meridianin C has not been reported previously. In this study, we demonstrate that meridianin C has strong growth suppressive and macropinocytosis inducing effects on YD‐10B human tongue cancer cells and the effects are mediated in part through regulation of DKK‐3 expression.

Aforementioned, meridianin C, D or G analogues/derivatives are anti‐proliferative in several types of human cancer cells, including breast (MCF‐7), cervix (HeLa) and leukaemia (MV4‐11).^21^, ^22^, ^23^ Here we initially screened four different human tongue cancer cell lines (YD‐8, YD‐10B, YD‐38 and HSC‐3) to see whether meridianin C treatment affects the cell survival. We found that meridianin C strongly and specifically reduced the survival of YD‐10B cells. However, the apoptotic parameters, including nuclear DNA fragmentation, population of sub G1, expression of procaspase‐9 (no expression of cleaved and active form of caspase‐9) and PARP cleavage, did not indicate apoptosis as the mechanism of action of meridianin C. Moreover, the microscopic and TEM analyses in this study showed that meridianin C rapidly induced cytoplasmic vacuoles/macropinosomes in YD‐10B cells. The SEM analysis further demonstrated, the ability of meridianin C to rapidly induce abundant long and thin membrane extensions that resemble lamellipodia on the surface of YD‐10B cells. These results strongly support the notion that the formation of macropinosomes induced by meridianin C in YD‐10B cells is the result of macropinocytosis. Meridianin C has previously been reported to inhibit Pim‐1, CDK‐1, CDK‐5, PKA, PKG, GSK‐3β and CK‐1 activity with IC_50_ values of 1.4, 3, 6, 0.7, 0.4, 2 and 30 μM, respectively.^23^, ^40^ There are no reports showing a relationship between cell survival or macropinocytosis induction and those kinases in cancer cells including YD‐10B cells. However, one can speculate that meridianin C's growth inhibitory and/or macropinocytosis inducing effects on YD‐10B cells may be through acting upon one or more of these kinases, which yet remain to be elucidated.

A notable finding of the present study is meridianin C regulation of DKK‐3 expression in YD‐10B cells. DKK‐3 is one of the DKK protein families and is known as a Wnt antagonist.^41^, ^42^ It is also recognized as a potential tumour suppressor, based on the facts that DKK‐3 expression is frequently down‐regulated in a wide array of malignancies.^43^, ^44^ Recent evidence indicates that DKK‐3 may also play additional roles in cancer cell survival. DKK‐3 knock‐down by siRNA in T24 human bladder cancer cells inhibited cell growth and induced macropinocytosis and autophagy.^16^ It also has been recently demonstrated that DKK‐3 is expressed in a subset of OSCC‐derived cell lines including HSC‐4.^33^ Moreover, in HSC‐4 cells, DKK‐3 knock‐down inhibited migration and invasion. These results imply that DKK‐3 may have a metastasis‐related oncogenic function in OSCC. Here we found DKK‐3 is highly expressed in YD‐10B cells and meridianin C suppresses its expression. To the best of our knowledge, this is the first study showing the ability of meridianin C to down‐regulate DKK‐3 in cancer cells. Of further importance, the present study revealed that siRNA‐based gene silencing of endogenous DKK‐3 led to partial accumulation of vacuoles in YD‐10B cells along with partial inhibition of the cell survival. Moreover, we herein have demonstrated that overexpression of DKK‐3 blocks the marcopinocytosis inducing property of meridianin C and leads to increase in cell survival against meridianin C treatment. Altogether, these findings show that DKK‐3 may have a role in controlling survival and macropinocytosis in YD‐10B cells and this is inhibited by meridianin C.

The reversibility of cancer cell growth inhibition after drug removal has clinical implications. In this study, we further evaluated the effects of meridianin C withdrawal on cell survival and macropinocytosis in YD‐10B cells. We found that meridianin C's anti‐survival and macropinocytosis inducing effects on YD‐10B cells were reversible. Several reports have recently described that hyper‐stimulation of macropinocytosis and/or disruption of normal macropinosome maturation associated with abnormal/massive accumulation of intracellular vacuoles leads to the cytotoxicity and/or nonapoptotic cell death of cancer cells.^45^, ^46^, ^47^ We would similarly speculate that the anti‐survival effect of meridianin C on YD‐10B cells is closely linked to this drug‐induced rapid and sustained accumulation of macropinosomes in the cells. We have similarly found that meridianin C can induce macropinocytosis (macropinosomes) in HSC‐3 cells, another human tongue cancer cell line, indicating that the meridianin C's macropinocytosis effects are not limited to YD‐10B cells. Further characterization of meridianin C's responses in YD‐10B cells compared to YD‐8 and YD‐38 cells, which are unresponsive to meridianin C, may further help to elucidate its mechanism of action.

We have recently synthesized a series of meridianin C derivatives, and demonstrated their anti‐proliferative activity in human leukaemia cell lines including MV4‐11.^23^ In the current study, we further compared the growth inhibitory and/or macropinocytosis inducing effects of meridianin C and its derivatives on YD‐10B cells. Although some of meridianin C derivatives had macropinocytosis inducing and growth inhibitory effects on YD‐10 cells, it was evident that their effects were much weaker than those induced by its parental compound meridianin C. These results indicate that meridianin C has a unique structural moiety leading to strong growth suppressive and macropinocytosis inducing activity in YD‐10B cells, which is distinct from other cancer cells.

In summary, meridianin C induces a significant level of macropinocytosis and selectively decreases the survival of YD‐10B cells in part through a reduction in DKK‐3. Although there are still important issues that remain to be resolved, including meridianin C's macropinocytosis inducing and anti‐tumour effects on animal models, our findings suggest that meridianin C is a potential novel target for metastatic human tongue cancer.

## ACKNOWLEDGEMENTS

This work was supported by the Korea Basic Science Institute (grant no. D37403) and the National Research Foundation of Korea (NRF) grant funded by the Korea Government (MSIP) (no. 2014R1A5A2010008).

## CONFLICT OF INTEREST

The authors declare that there is no conflict of interest.

## AUTHORS CONTRIBUTION

N.‐S.P., Y.‐K.P., H.‐R.C., J.‐H.B., K.N.M., M.R., J.K., M.N. and A.K.Y performed experiments. J.L., J.‐S.C. and B.‐C.J. designed the research study and supervised the study. V.S.H., D.B.‐B. and B.‐C.J. wrote the manuscript. J.L., J.K., M.N., K.‐B.L., I.‐S.J., D.B.‐B. and B.‐C.J. analysed the data.
